# Reversal of partial botulinum toxin A resistance following Janus kinase inhibition with tofacitinib: A case series

**DOI:** 10.1016/j.jdcr.2025.08.030

**Published:** 2025-09-09

**Authors:** Simona Abou Tayeh, Nakhle Ayoub

**Affiliations:** a School of Medicine and Medical Sciences, Holy Spirit University of Kaslik, Jounieh, Lebanon; b Dermatology Department, Hôtel-Dieu de France, Beirut, Lebanon

**Keywords:** abobotulinum toxin A, botulinum toxin type A, incobotulinum toxin A, JAK inhibitors, onabotulinum toxin A, resistance, tofacitinib

## Introduction

Botulinum toxin (BT) is a highly potent neurotoxin produced by *Clostridium botulinum* that inhibits acetylcholine release from presynaptic cholinergic neurons.^1^ *Clostridium botulinum* is a spore-forming, gram-positive, anaerobic bacterium that has 7 serotypes, A to G, and is prepared in 4 types for practical use. There are 3 forms of serotype A and 1 form of serotype B. All 3 serotypes A are approved globally for aesthetic purposes. These serotypes work through the inhibition of a set of proteins, the soluble N-ethylmaleimide-sensitive factor attachment protein receptor complex, causing flaccid paralysis of the target muscle. The clinical effects derive from minimizing muscle tension and enhancing muscle relaxation. The most potent and commonly used toxin type is A,^2^ and its injection has remained the most popular aesthetic procedure since 1999.^3^

Many factors are involved in therapy failure. Repeated exposure to BT can lead to the formation of antibodies that neutralize the toxin’s effects, resulting in secondary resistance. Variations in the manufacturing process, such as the type of bacterial strain used and the excipient, can also contribute to resistance.^4^ Inadequate dosing or an improper frequency of injections can also cause suboptimal results, as low toxin levels may not produce the desired therapeutic effects. Furthermore, cross-resistance can occur when antibodies developed against 1 formulation affect the efficacy of others. Chronic exposure to or overuse of BT can also lead to tolerance because the muscle becomes less responsive due to changes in receptor sensitivity.^5^ When BT resistance occurs, it is associated with long-term clinical unresponsiveness, and except for the shift from 1 formulation to another, there is no therapy available to reverse resistance.

Complete and partial resistance to BT refers to different degrees of nonresponsiveness to the toxin’s effects. Complete resistance means the patient shows no response to BT injections, while partial resistance indicates a diminished or reduced response compared to previous treatments, requiring higher doses or more frequent injections for similar effects. We report here 2 cases of secondary partial resistance to BT.

## Case report

A 47-year-old woman with no significant medical or surgical history presented to our clinic after experiencing suboptimal results from multiple BT injections over the previous years. Her primary concern included prominent facial wrinkles, especially in the forehead, glabella, and crow’s feet. Prior to her presentation, she had undergone BT injections multiple times, including 6 cycles of Botox (Allergan), 2 cycles of Dysport (Ipsen Limited), 2 cycles of Neuronox (Medytox), a single injection of Xeomin (Merz Pharmaceuticals GmbH), and 1 cycle from an unlicensed tattoo artist. The patient’s prior BT treatments were administered at regular 6-month intervals for most of the treatment course. Notably, the second cycle of Neuronox and the single Xeomin injection were administered at 2 different clinics only 10 weeks apart, an unusually short interval compared with her previous cycles.

Despite these procedures, she experienced partial responsiveness and reported a shorter duration of efficacy. Her initial response to Botox and Dysport was satisfactory but became progressively shorter with each subsequent session. Her response to Xeomin and Neuronox was limited to only 3 weeks. No significant adverse reactions were noted aside from the brief duration of effect. The patient was diagnosed with secondary BT resistance, likely due to the development of neutralizing antibodies, although no testing for antibodies was available. In an attempt to reverse resistance, we initiated treatment with tofacitinib, a Janus kinase (JAK) inhibitor, at a dose of 10 mg daily. After 2 weeks of continued tofacitinib therapy, she received another Botox injection. She experienced substantial improvement in the duration and efficacy of the treatment, which lasted for 3 months, in comparison with the 3-week response previously reported (Table I). Four months after the injection, the patient underwent another cycle of BT. Tofacitinib was reinitiated for that session as a 1-month course, began 15 days prior to the injection, and was maintained for 15 days postinjection. The clinical improvement observed following this combined approach was maintained for at least 4 months after the repeat treatment.

**Table I:** Case 1: a 47-year-old woman

Several years before presentation	Initiation of BT injections with suboptimal results Received 6 cycles of Botox (Allergan) and 2 cycles of Dysport separated by a regular 6-mo interval Then received 2 cycles of Neuronox, 1 of Xeomin and 1 injection from an unlicensed tattoo artist. The second cycle of Neuronox and the single Xeomin injection were administered only 10 wk apart.
Response to Botox and Dysport	Satisfactory initially but became progressively shorter with each subsequent session.
Response to Neuronox and Xeomin	Limited to only 3 wk
At presentation (wk 0)	Initiation of tofacitinib, a Janus kinase (JAK) inhibitor, at a dose of 10 mg daily
After 2 wk (wk 2 or 14 d)	Received 1 cycle of Botox
Post injection	Marked improvement in efficacy and duration (response lasting 3 mo)
4 mo after the injection	1 cycle of Botox with tofacitinib administered 15 d prior to the injection and was maintained for 15 d postinjection. Clinical response was maintained for at least 4 mo.

*BT*, Botulinum toxin.

Building on the findings of the previous case, the following case provides additional evidence for the efficacy of tofacitinib in restoring BT responsiveness.

A 40-year-old woman who presented for BT injections reported a history of resistance to botulinum toxin A (BT-A) formulations, including Dysport (twice), Botox (Allergan, once), and Xeomin (once). Each treatment was associated with transient efficacy lasting 1 to 3 weeks. Her medical history was significant for alopecia areata for the past year, which had initially responded to topical minoxidil 5% and clobetasol propionate cream. However, as her condition progressed, these therapies became ineffective. Methotrexate (15 mg weekly) was prescribed and used for 2 months, but the patient experienced no clinical improvement, leading to its discontinuation. She had no other significant medical history or comorbidities. Three weeks prior to the current BT-A injection, the patient was prescribed 10 mg tofacitinib daily at another clinic for her alopecia areata. This intervention was initiated following a diagnosis of refractory alopecia areata based on clinical findings.

Her prior BT-A injections were appropriately spaced and administered by qualified practitioners, yet each formulation resulted in minimal therapeutic benefit. Physical examination revealed no findings suggestive of systemic autoimmune disease, apart from alopecia areata. Given the patient’s recent initiation of tofacitinib, it was hypothesized that immune modulation might address her suspected resistance to BT-A. She effectively underwent an injection of 50 units of Botox (Allergan) for the treatment of glabellar lines and reported a robust clinical outcome, with significant improvement noted within 5 days of injection. The response was maintained for more than 3 months without requiring additional touch-up injections to contrast with her prior experience with BT-A formulations (Table II).

**Table II:** Case 2: a 40-year-old woman

Several years before presentation	Initiation of BT-A injections with suboptimal results Received 2 cycles of Dysport and 1 cycle of Botox. Then received 1 cycle of Xeomin.
Response to each cycle	Transient efficacy lasting 1 to 3 wk
3 wk prior to her BT-A injection	10 mg tofacitinib daily was prescribed for her alopecia areata.
At presentation	50 units of Botox was administered
Post injection	Marked improvement within 5 d, sustained response >3 mo

*BT-A*, Botulinum toxin A.

In this case, the patient’s resistance to multiple BT-A formulations suggests immunogenicity as the underlying cause. Although tofacitinib has been established as an effective therapy for alopecia areata, its role in reversing BT-A resistance has not been reported. It is plausible that the immunomodulatory effects of tofacitinib reduce the production of neutralizing antibodies or alter immune pathways, contributing to patients’ prior resistance.

## Discussion

These 2 cases underscore the need for further investigation into the interplay between immune modulation and BT-A resistance. For patients with suspected immunogenic resistance, therapies targeting the immune system may offer a novel avenue for restoring responsiveness to BT.

Tofacitinib is a Janus-associated kinase inhibitor approved by the Food and Drug Administration for the treatment of adult patients with moderate to severe rheumatoid arthritis who have an inadequate response or intolerance to methotrexate therapy.^6^ It has been prescribed for alopecia areata because of its off-label benefit. By inhibiting JAKs, tofacitinib prevents the phosphorylation and activation of signal transducers and activators of transcription (STATs). The proposed mechanism by which JAK inhibitors may reverse BT-A resistance centers on their ability to modulate key pathways involved in both B-cell and T-cell immune responses. The JAK-STAT signaling pathway is involved in the transcription of cells involved in hematopoiesis and immune cell function. JAK inhibitors, such as tofacitinib, work therapeutically by blocking the JAK-STAT signaling cascade, which transduces signals from a wide array of cytokines. Tofacitinib acts by suppressing the overactive T helper (Th) 2 immune response, as interleukin (IL) 4 is crucial for stimulating the Th2 immune response and the release of cytokines such as IL-4, IL-5, IL-10, and IL-13^7^—all of which play critical roles in B-cell activation, plasma cell differentiation, and antibody production. By attenuating these signals, JAK inhibition can reduce class-switch recombination and decrease the generation of neutralizing antibodies. Additionally, JAK inhibitors suppress Th cell differentiation, particularly Th1 and Th17 subsets, which are essential for driving cytotoxic and proinflammatory responses.^8^ T-cell support is essential for effective germinal center formation, affinity maturation, and the generation of plasma cells; therefore, suppression of T-cell activation and cytokine signaling through JAK inhibition may secondarily impair B-cell differentiation and the production of high-affinity, class-switched antibodies. While direct evidence in the context of BT-A resistance is lacking, the immunomodulatory properties of JAK inhibitors provide a plausible rationale for their observed clinical effect in patients with suspected immune-mediated toxin unresponsiveness.

Resistance to BT remains a rare but challenging complication that may be attributed to the development of neutralizing antibodies. Primary resistance to BT has rarely been reported. Pirazzini et al suggested that primary nonresponsiveness may be caused by pre-existing botulinum neurotoxin-neutralizing antibodies from unnoticed botulism or mutations in receptors that modify the toxin’s interaction sites.^9^ They discussed the possibility that mutations in the synaptic vesicle protein SV2, which serves as a receptor for botulinum neurotoxin type A (BNTA), could hinder the ability of the toxin to bind and exert its effect.

From an immunologic standpoint, the induction of a neutralizing antibody response against BT-A hinges on 2 key checkpoints. The first occurs at the level of dendritic cells, which use pattern recognition receptors such as Toll-like receptors to assess whether an encountered antigen poses a potential threat. Upon recognizing danger-associated molecular patterns, dendritic cells engulf the antigen, migrate to lymph nodes, and present peptide fragments via major histocompatibility complex molecules to naïve CD4^+^ Th cells. The second checkpoint involves the activation of these T cells, which, upon recognizing the antigen as foreign and receiving co-stimulatory signals, undergo clonal expansion and facilitate B-cell activation and class-switch recombination.^3^ This tightly regulated, hierarchical process underscores the importance of both innate and adaptive immune recognition in antibody formation. Modifiable factors such as the purity of the BT-A formulation, the total dose delivered, and the frequency of injections all influence the likelihood of triggering this immunologic cascade. High doses or repeated administration increase the probability of the antigen being perceived as immunologically “dangerous.”

Indeed, several factors may contribute to a suboptimal or absent response to BT treatment. These include the injection technique, disruption of the cold chain during storage or transport, compromised toxin integrity, improper dilution, inadequate dosing, patient-related factors such as unrealistic expectations, and the development of resistance.^10^ Toxin conditions can be altered under certain conditions, such as exposure to alcohol during skin disinfection, the application of ice packs immediately postinjection (which can cause vasoconstriction and reduce toxin absorption), improper storage like freezing the reconstituted toxin (which may cause thermal degradation), or vigorous agitation of the vial after dilution.^4^

To date, there is no known treatment for BT resistance. Its management involves a multifaceted approach aimed at optimizing efficacy while minimizing immunogenic risk. Management focuses on optimizing dosing strategies, improving muscle targeting (often with electromyography or ultrasound guidance), and extending injection intervals to reduce neutralizing-antibody formation. When clinical resistance arises, switching serotypes or formulations is considered. Prophylaxis includes using the lowest effective dose, patient education, and adhering to recommended intervals (6 months, minimum 3), with any touch-ups limited to 1 session within 1 week.^11^

Even without prior exposure, primary resistance can occur. A case series published in the *Aesthetic Surgery Journal* reported a 6.25% primary-resistance rate in noncosmetic treatments, suggesting pre-existing antibodies or receptor mutations.^12^

A 15-case series across 12 sites demonstrated alternative treatment options after a diagnosis of resistance to BNTA.^13^ Among the patients only exposed to alternative BT-A, 2 patients (13.3%) overcame resistance. Of 7 patients transitioned to botulinum neurotoxin type B (RimabotulinumtoxinB), 85.7% responded; this was significantly higher than the response rate to another BNTA (*P* = .0023) (Fisher’s exact test).

Tofacitinib, the first JAK inhibitor approved for rheumatoid arthritis, has been extensively studied, offering substantial evidence regarding its efficacy and safety profile. Major trials, notably the Oral Rheumatoid Arthritis series, have been instrumental in defining its clinical role. Like other agents in its class, tofacitinib’s safety is primarily evaluated in relation to serious infections, malignancies, and viral reactivation. Clinical trials have consistently shown an increased risk of serious infections and a possible rise in certain cancers compared to conventional disease-modifying antirheumatic drugs, likely due to its immunosuppressive effect on the JAK-STAT pathway.^14^ Tofacitinib is also known to cause laboratory abnormalities, such as altered lipid profiles, elevated liver enzymes, and herpes zoster reactivation necessitating regular biochemical surveillance during treatment.

Given these safety concerns, especially in contexts where the goal is quality-of-life improvement rather than disease survival, a thoughtful risk-benefit assessment becomes crucial. In aesthetic indications, where the impact is primarily functional or psychosocial, the threshold for acceptable risk must be lower. Thus, patient selection, informed consent, and close monitoring are essential when considering off-label JAK inhibitor use. Despite these caveats, our case series introduces a novel and potentially impactful application of JAK inhibition that warrants further exploration.

While the temporal association between tofacitinib initiation and restored responsiveness to BT-A is compelling, several limitations warrant careful consideration. First, serologic testing for neutralizing antibodies was not performed in either case, preventing definitive confirmation of an immunologic basis for resistance. To our knowledge, no clinical laboratory in Lebanon currently offers testing for BT neutralizing antibodies. Spontaneous recovery of BT-A sensitivity or placebo-related effects remain plausible alternative explanations. Moreover, patients’ expectations can significantly influence their perception of BT efficacy, potentially leading to subjective nonresponse despite effective treatment. The placebo effect during the initial treatment cycles often amplifies the perceived efficacy of BT.

These findings illustrate the potential role of JAK inhibitors, such as tofacitinib, in reversing BT resistance. While many studies have suggested that JAK inhibitors may modulate the immune response, including antibody production, their ability to reverse BT resistance is not well documented in the current literature. The patients’ response to tofacitinib highlights the possible use of this medication as a therapeutic option for patients who exhibit resistance to BT, offering a novel approach to manage this clinical challenge. Although further studies are needed—ideally with immune profiling and serologic testing—to establish the role of JAK inhibitors in BT resistance, these cases provide compelling evidence for their potential use in restoring the therapeutic effects of BT in resistant patients.

## Conflicts of interest

None disclosed.
